# Fall risk in an active elderly population – can it be assessed?

**DOI:** 10.1186/1477-5751-6-2

**Published:** 2007-01-26

**Authors:** Uffe Laessoe, Hans C Hoeck, Ole Simonsen, Thomas Sinkjaer, Michael Voigt

**Affiliations:** 1 Center for Sensory-Motor Interaction (SMI), Aalborg University, Fredrik Bajers vej, Aalborg, Denmark; 2 Center for Clinical and Basic Research, Hobrovej, Aalborg, Denmark; 3 Northern Orthopedic Division Aalborg Hospital, part of Aarhus University Hospital, Hobrovej, Aalborg, Denmark

## Abstract

**Background:**

Falls amongst elderly people are often associated with fractures. Training of balance and physical performance can reduce fall risk; however, it remains a challenge to identify individuals at increased risk of falling to whom this training should be offered. It is believed that fall risk can be assessed by testing balance performance. In this study a test battery of physiological parameters related to balance and falls was designed to address fall risk in a community dwelling elderly population.

**Results:**

Ninety-four elderly males and females between 70 and 80 years of age were included in a one year follow-up study. A fall incidence of 15% was reported. The test battery scores were not different between the fallers and non-fallers. Test scores were, however, related to self-reported health. In spite of inclusion of dynamic tests, the test battery had low fall prediction rates, with a sensitivity and specificity of 50% and 43% respectively.

**Conclusion:**

Individuals with poor balance were identified but falls were not predicted by this test battery. Physiological balance characteristics can apparently not be used in isolation as adequate indicators of fall risk in this population of community dwelling elderly. Falling is a complex phenomenon of multifactorial origin. The crucial factor in relation to fall risk is the redundancy of balance capacity against the balance demands of the individuals levels of fall-risky lifestyle and behavior. This calls for an approach to fall risk assessment in which the physiological performance is evaluated in relation to the activity profile of the individual.

## Background

Amongst elderly people bone fractures in relation to falls are a frequent phenomenon. These accidents are often associated with physical decline, negative impact on quality of life and reduced survival [1]. Fall risk has been related to a number of factors such as history of falls, muscle weakness, gait deficit, balance deficit, use of assistive device, visual impairment, mobility impairment, fear of falling, cognitive impairment, depression, sedentary behavior, age, number of medications, psychotropic/cardiovascular medications, nutritional deficits, urinary incontinence, arthritis, home hazards and footwear [2,3].

The natural ageing process combined with inactivity can gradually lead to decreased physical performance with the result that many elderly are at increased risk of falling [4]. Several studies have found that interventions can reduce the fall rate in an elderly population [5]. Different interventions have been suggested ranging from initiatives to ensure a safer environment to specific methods of training of the individual [6-9]. Part of the deterioration in physiological capacity seems to be due to a lack of stimulation and training and this can be addressed by exercise. Exercises comprising balance training and strength training have proven the most effective in relation to reduction in fall incidence [8].

The very old and fragile elderly have an increased risk of falling and it has been suggested that all people over 80 years of age should be offered exercise training regardless of risk factor status [10]. In line with this it is relevant to focus on the group of elderly under 80 years of age to identify the individuals in this group who would need balance training. In the current study it was decided to include community-dwelling elderly aged 70 to 80 years.

The identification of individuals at risk of falling is not a trivial matter. Many different physiological performance tests are believed to be sensitive to fall risk. Several research groups have investigated combinations of tests to produce test batteries addressing fall risk in the elderly [11-13]. The reported prediction rates vary a great deal according to the characteristics of the elderly populations included in the different studies.

The present study covers a generally active population of elderly. Within this group it is believed that fall risk assessment should include dynamic and attention demanding balance tests [14,15]. It has been shown that increased gait variability relates to fall risk [16]. If gait is solely executed in relation to sensory feed-back, each step will include a great deal of balance adjustment and this will lead to an uneven gait pattern. Stable gait calls for motor planning in order to allow a feed-forward strategy that adjusts the next step in an appropriate way [17]. This means that stable gait requires a proactive dynamic postural control and orientation in space. An assessment of gait is therefore relevant in this context. The vision providing the information for postural planning must naturally also be tested [18]. A dual task testing approach, has been proposed to reveal early signs of insufficient postural control [19]. In a dual task situation the subject must perform a cognitive task in parallel to a motor task. This occurs frequently in daily life situations and poor dual task performance seems to be related to fall risk [20]. A test battery evaluating fall risk in a population of active elderly should therefore include these aspects in the tests. Muscle strength is a strong predictor of fall and a test of muscle strength must be relevant in such a test battery along with some sort of test of general physical function [2,21]. Assessments of the ability to maintain a standing position by equilibrium reactions and the ability to make base of support reactions are relevant as indicators of basic balance aspects [22-24]. Nine specific tests, including dynamic tests, were selected for the test battery in order to cover these different aspects of physical performance which could be related to fall risk.

The purpose of the current study was to develop a tool to identify community dwelling elderly individuals in risk of falling. The study should evaluate the capability of a new test battery to predict fall incidence in an active elderly population between 70 and 80 years of age.

## Results

The study population of elderly had a mean age of 73.7 years (sd. 2.9) and the proportion of males was 26%. Fifteen percent of this population experienced at least one fall during the one-year follow-up period. The groups of fallers and non-fallers were not significantly different regarding age and measures of self estimated health, physical activity level and balance confidence (table 1). There were relatively more males in the non-fallers group (14% versus 27%) and more individuals reported non-balance-related illness and balance-related illness in the fallers group (43% versus 29% and 55% versus 20%). None of these differences were statistically significant.

**Table 1:** Group characteristics of fallers and non-fallers

	Fallers (n = 14)		Non-fallers (n = 80)	
Age	73.0	(2.9)	73.8	(2.9)
BMI^a^	26.8	(3.2)	27.3	(4.7)
Health ^b^	4.4	(0.7)	4.3	(0.5)
PASE ^c^	125	(55)	126	(48)
ABC ^d^	91	(8)	90	(13)

^a ^Body Mass Index; ^b ^Self estimated health on a scale from 1–5, with 1 being very bad and 5 being very good; ^c ^Physical Activity-based Scale for the Elderly; ^d ^Activity-specific Balance Confidence Scale. Values are mean and standard deviation ().

The test raw scores for fallers and non-fallers are presented in table 2. In only one of these individual tests of the test battery, a statistically significant difference was found between fallers and non-fallers. This regarded test no.1 on "balance in standing position", (p < 0.05).

**Table 2:** Test scores for fallers and non-fallers

	Test focus	Outcome	Fallers	Non-fallers
1	Standing balance	Performance scale (0–6)^a^	4.5 (3–5)	5.0 (2–5.5)
2	Stepping ability	Time required (s)	9.8 (1.3)	9.8 (3.2)
3	General function	Time required (s)	8.8 (2.2)	8.8 (1.9)
4	Reaction time	Averaged time to step (s)	0.82 (0.14)	0.89 (0.21)
5	General leg strength	Time required (s)	23.9 (7.6)	24.5 (9.1)
6	Dual task	Speed reduction (%)	35 (30)	30 (27)
7	Gait variability	Autocorrelation (no unit)	0.85 (0.05)	0.84 (0.06)
8	Gait cadence	Steps per second	1.7 (0.1)	1.7 (0.1)
9	Vision	Acuity/contrast/field (0–7)^a^	5 (4–7)	6 (2–7)

Test scores of the nine tests of the test battery presented by mean and standard deviation () or ^a ^median and range ().

**Figure 1:**
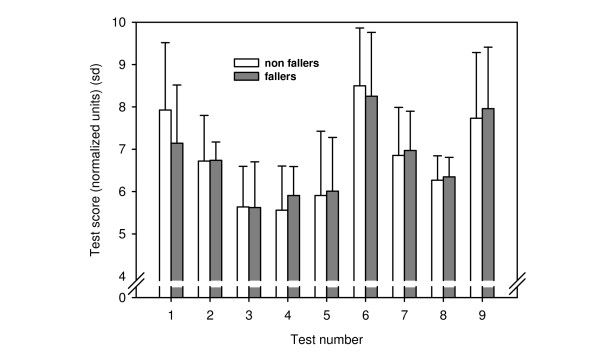
**Test scores from the nine tests in the test battery**. Mean scores and standard deviations are presented in normalized units on a 0–10 scale with higher values indicating better performance. Test numbers are referring to: 1) Standing balance, 2) Stepping ability, 3) General physical function, 4) Reaction timer, 5) Leg strength, 6) Dual task, 7) Gait variability, 8) Gait cadence, 9) Vision.

To evaluate the common product of the tests as a test battery, the scores were converted into 0–10 scales with higher values indicating better performance. The converted scores of the tests are seen in figure 1. It can be seen, that the group of fallers actually had a higher mean score than the non-fallers in some of the tests.

**Figure 2:**
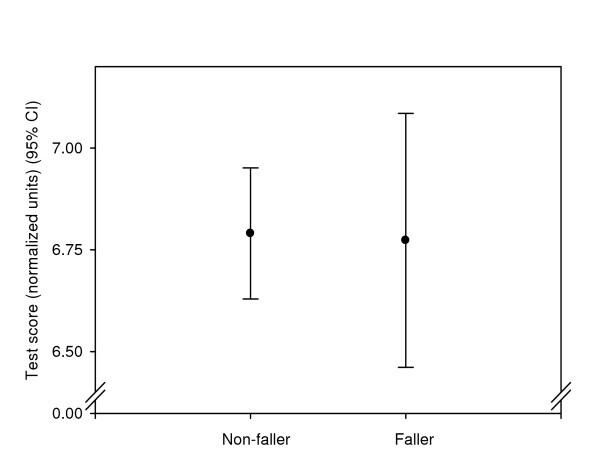
**Test battery scores for fallers and non-fallers**. Mean test battery scores with 95% confidence intervals are given in normalized units on a 0–10 scale with higher values indicating better performance. No significant difference was found between faller and non-faller group.

For each subject the test scores were averaged into a test battery score. No statistically significant difference between fallers and non-fallers was seen. The score was 6.8 (0.6) for the fallers and 6.8 (0.7) for the non-fallers (figure 2).

When choosing 0.5 (5%) to be the least clinical relevant difference in the test battery score, the power of the study was 85.4%. At a power of 80% the equivalent least statistically significant difference in test battery score was 0.46.

In a logistic regression the tests showed no predictive capability in relation to fall incidence. Neither did the combination of the tests as a test battery predict who fell in the one-year follow-up period (OR = 0.98; p = 0.97).

**Figure 3:**
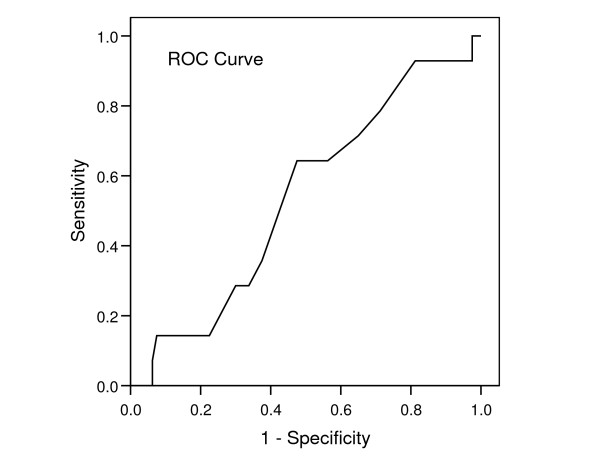
**Receiver operating curve**. A ROC curve is a plot of the true positive rate against the false positive rate for the different possible cutoff points of the test battery. In this way the trade off between sensitivity and specificity is illustrated and an optimal cut-off value can be suggested. An ideal curve reflecting high sensitivity and high specificity would be positioned towards the left and the upper border with an area under the curve approaching 100%. The presented curve shows no tendency to perform this way.

No clear cut-off point could be suggested for the test battery score in relation to falls (figure 3). An optimal fall prediction was obtained by a cut-off value of 6.9 producing a sensitivity of 50% and a specificity of 43%. The corresponding positive and negative prediction rates were 13% and 83% respectively.

### Alternative measures

Test battery scores correlated significantly, with low correlation values, to the scores in questionnaires on self estimated health, activity level and balance confidence. The Spearman's correlation values were 0.33, 0.44 and 0.37 respectively (p < 0.001).

**Figure 4:**
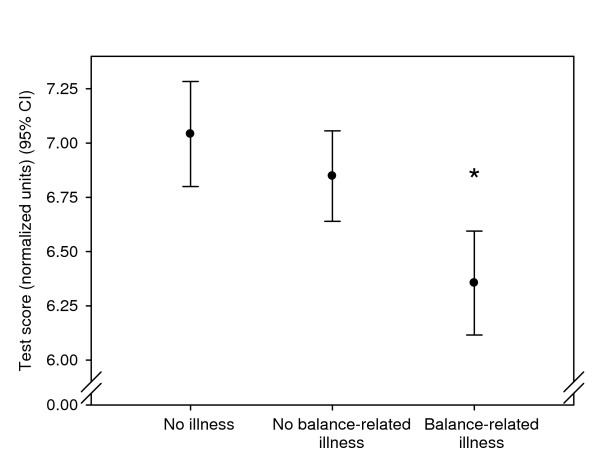
**Test scores related to self reported illness**. Mean test battery scores with 95% confidence intervals are given in normalized units on a 0–10 scale with higher values indicating better performance. The groups reporting illness had lower scores in the test battery. Significant differences were found between the "balance-related illness" group and the two other groups. * (p < 0.01)

Self reported illness was grouped into three categories according to the influence on balance and gait: no illness (n = 24), illness which was not regarded to be balance related (n = 50) and balance related illness (n = 20). The distribution of reported balance-related illness was not significantly different between fallers and non-fallers (p = 0.67). When the elderly population was divided into three groups according to balance-related illness statistically significant differences were found in test battery scores between the "balance-related illness" group and the two other groups, but not between the "no illness" and the "no balance-related illness" groups (figure 4).

## Discussion

The population included in this study had a fall incidence of 15%, which must be regarded as a low percentage for this age group. It has previously been reported, that the proportion of community-dwelling elderly sustaining at least one fall over a one-year period varies from 28 – 35% in the +65-year age group to 32 – 42% in the +75-year age group [25]. Four subjects reported recurrent falls. Recurrent falls are even more indicative of balance related fall risk, but this incidence was too small to be evaluated. The subjects were contacted at half year intervals and this might have introduced a recall bias. If the subject failed to recall a fall event in the preceding six month an underreporting of fall incidence would occur. During the interviews however we experienced no indications of recall problems. These elderly were cognitively well functioning and a fall accident seemed to be an event which was easily remembered. The low fall incidence is likely to be a natural consequence of the selection of the study population. The community-dwelling elderly attending activities at elderly centres might belong to a relatively healthier group of elderly. The fall risk prediction is probably more challenging but not less relevant in this population.

In spite of the low fall incidence the power of the study was strong and would have revealed a clinically relevant difference of 0.5 in test battery score between the two groups. It is, however, always an open choice to decide when a difference is clinical relevant. In this population the test score had a standard deviation of 0.7. A difference of 0.5 (equivalent to 5% on the 0–10 scale) was therefore regarded as a relevant but also very demanding limit of clinical relevance. In respect to this level of difference the study produced a power of 85%. For a conventional choice of a power of 80% a significant decrease in score for the fallers should have been 0.46 on the 0–10 scale.

In relation to the testing session the participants were also interviewed about health problems. This was mainly done for an in- or exclusion purpose, but it also provided the possibility to group the included participants into three categories in relation to health. It was seen that balance-related illness did not relate to fall incidence, but it did relate significantly to the test battery score (figure 4). This indicated the test battery did in fact reflect the physiological status of the participants.

When comparing the group of fallers with non-fallers, no statistically significant difference was seen in fall related physiological performance and balance as scored by the test battery. This naturally implicated a poor capability of the test battery to predict falls. Despite the fact that the individual tests in the test battery all addressed physiological parameters, which had previously been shown to relate to fall risk, these tests performed poorly as fall predictors in this population of elderly.

In a case control evaluation on a subset of this study population the same test battery produced better discrimination rates [26]. Two age-matched subgroups of 35 women with and 36 women without a history of falling within the previous two years were compared. Significant differences could be found when comparing test battery scores between these groups. The fallers had an average score of 6.5 (SD 0.9) on the normalized 0 – 10 scale whereas the non-faller group scored 7.0 (SD 0.4). The difference of 0.5 was statistically significant (CI: 0.2 – 0.8) (p < 0.01). In this analysis the test battery discriminated between fallers and non-fallers with a sensitivity of 71% and a specificity of 58%. When validating a test in a case-control study, the inclusion of selected fallers as "cases" often produces good discrimination rates. Unfortunately, this is not necessarily the case when the same test is included in a cohort study on community-dwelling elderly. This tendency has also been seen in other studies on fall risk [11,27]. The necessity for prospective studies must be underlined in the evaluation of tests meant for prediction. In prospective studies however another risk of bias occurs. The elderly will become aware of potential deficits in their balance performance during the testing session, and this information might influence their behaviour during the follow-up period.

### Fall risk factors

**Figure 5:**
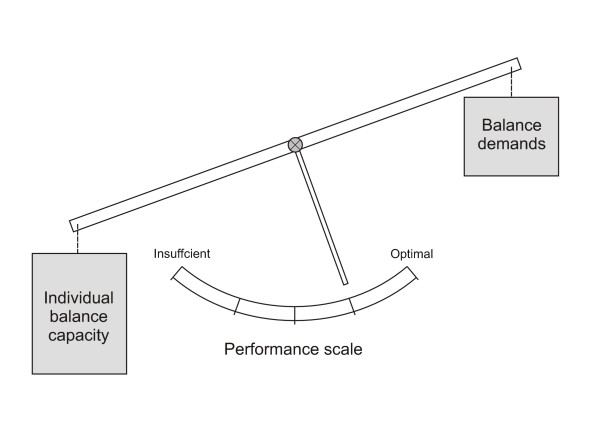
**Balance performance model**. This is a model to illustrate the interaction between balance capacity and balance demands. The interaction is reflected in an outcome which is measured on a given scale. A redundancy of balance capacity in relation to balance demands will ensure a good balance performance. On the contrary can increased balance demands or loss of balance capacity result in insufficient performance

A major problem, when predicting fall risk, is the multi-factorial mechanisms of falls. The influence of environmental factors and the difficulty in daily tasks performed have to be considered as well as the individual physiological factors [28]. To be able to cope well in daily-life situations the balance demands in the environment and in the tasks performed must be matched by the balance capacity of the elderly. These reflections are illustrated in figure 5. This figure shows the interaction between the individual balance capacity and the challenges offered by balance task and context. The interaction between these factors is related to a balance performance which is reflected as an outcome on the performance scale. As an example, when walking on an icy surface the increased "weight" on the balance demand side will be reflected on a "gait speed scale". Likewise it will be reflected in the performance when illness or age decrease the "weight" on the balance capacity side and this is outbalanced by the demand of standing on one leg with eyes closed.

Very fragile persons or individuals with a poor postural control might be very well aware of their balance lacking status. They will probably try not to challenge themselves beyond their limits and therefore, in spite of their low physical capacity, they might not be in high risk of falling.

Other individuals, who are healthy, fit and displaying good balance capacities, could live very active lives (outdoor walking in all kinds of weather, dancing and attending sporting activities etc.). From time to time these persons might challenge themselves beyond their limits and thereby be at increased risk of falling.

In telephone contacts during the follow-up period of this study these factors were recognized. Non-fallers would often explain that they were concerned about their performance. For example, they would avoid challenging their balance capacity by limiting gait speed or avoiding inclement weather and subsequent fall risks. In contrast to this, many of the fallers indicated that the fall had occurred during fall risky conditions. This could be falls playing soccer with the grandchildren, walking down an icy path in the forest or when influenced by alcohol.

In this study, the test battery assessed fall risk by evaluating solely the physical capabilities of the individual but in relation to fall risk the critical factor is, whether the balance capacity of the individual matches the individual balance demands. In fact, Gregg et al. (1998) described a U-shaped relationship between physical activity level and fall incidence (i.e. colles fractures) amongst elderly (+65 years of age). This implied that both sedentary and very active elderly were more at risk than average [29]. A redundancy of balance capacity is necessary to face challenges at the individual's relevant activity level. This redundancy was apparently lacking amongst the sedentary elderly due to poor performance and amongst the very active elderly due to excessive challenges.

The test battery score correlated against self-estimated health score, physical activity score and balance confidence score. Although these findings were significant, it should be noted that the correlations were not very high. A low physical capacity is not necessarily reflected in low physical activity level, low balance confidence or low health estimation. In fact, the neglect of poor physical performance level could lead to a relatively risky behaviour which again might lead to a higher fall risk. In such a case, it would be relevant to lead the individual to an awareness of the lacking balance capacity. A problem with this approach is, however, that it could also lead to anxiety and inappropriate restrictions in the physical activity.

**Figure 6:**
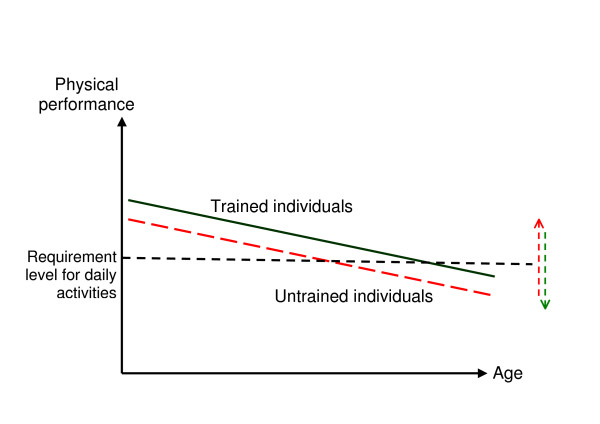
**Illustration of physical capacity and age**. An illustration of the normal deterioration of physical performance related to age. A well trained individual (thick green line) might deteriorate in parallel with the untrained individual (dashed red line) but they would cross the level at which they cannot face the challenges of daily life at a very different time in their life. Small arrows indicate that this level is individual.

To avoid falling it is necessary to have the physiological capacity to negotiate the threatening balance demands of a given task and context. For natural reasons the physiological capacity deteriorates gradually with age [30,31]. This makes it even more relevant to keep this capacity at its peak in accordance with the age-related expected performance. A classic way of illustrating this approach is given in figure 6. It shows that the deterioration will lead to a point, where the level of the physical requirements for normal daily activity is crossed but it also shows that the age corresponding to this crossing point is very much influenced by the individual starting point and maintenance of physical performance [32]. The figure also illustrates that the individual physical performance requirements for daily activities not necessarily are set at a fixed level.

### Perspectives

In this study it was not possible to identify individuals at increased fall risk amongst the active elderly between 70 and 80 years of age. In relation to fall prevention in this group of elderly subjects, we believe that it is not relevant to use general fall risk screening in order to target the balance training at selected individuals. This leads to the pragmatic approach that all elderly should be offered physical exercise to keep or restore the best possible redundancy in balance capacity.

General programs maintaining/improving gait performance and balance would appear to be worthwhile. Exercise must be regarded as a natural part of the daily life for the elderly. This is seen in some Asian societies where "Tai Chi" often is practiced as a daily routine also for the elderly. In some countries "Nordic walking" (where special sticks are used for walking) has become a popular way of outdoor exercise amongst the elderly. Both the individual and the society must take actions to facilitate physical exercise but also manufacturers of exercise tools should accept the challenge of developing equipment designed especially for the elderly.

Elderly people experiencing the first signs of balance deterioration might furthermore benefit from a clinical consultation on possible needs for specific exercises and adjustments of risky lifestyle to increase the balance redundancy.

Future studies on fall risk face the challenge of addressing both sides of the model illustrated in figure 5. The balance demand as well as the balance capacity has to be evaluated in order to estimate the balance redundancy. A less fragmented or dualistic approach which includes psychosocial factors and interactions with the environment might lead to new measuring scales for evaluating this redundancy.

## Conclusion

The physiological balance capacity can be addressed by tests related to balance and fall risk. However, falling is a complex phenomenon of a multi-factorial nature with associations to a fall-risky lifestyle. In any given situation redundancy in physiological capacity is crucial in order to negotiate balance threatening demands.

The results from this study support the view that fall risk cannot be predicted in a healthy and active elderly population by solely assessing physical performance. This calls for an approach to fall risk assessment in which the physiological performance is evaluated in relation to the activity profile of the individual.

## Methods

### Population

The study was designed as a cohort study with a one year follow-up period. It was conducted in a population of community dwelling healthy elderly from 70 to 80 years of age. The elderly were invited to participate in the study by announcements at senior community centers and by verbal contacts. A population of 101 elderly people was tested with the test battery. Five of these elderly were excluded due to the exclusion criteria. Two out of the 96 included participants were lost to follow-up because of severe illness or death.

The elderly were excluded if they reported any of the following: a) major musculoskeletal disorder; b) significant pain that limited daily functions; c) dependence on gait auxiliaries; d) ear infection within two weeks prior to the test; e) fall within one month prior to the test; f) dependence on special care to stay in community; g) known uncorrected visual or vestibular problems or h) cognitive impairment (Mini Mental State Examination (MMSE) < 23) [33].

Informed consent was obtained from all participants prior to inclusion in the study. The protocol was approved by the Ethics Committee of Viborg and Nordjyllands Counties.

### Procedures

The elderly were tested at local senior citizens community centres. The participants were introduced to each test in the test-battery by a demonstration following which they were allowed to do a pre-trial test. The participants were interviewed about age, height, weight, fall history and health problems. In spite of the exclusion criteria seventy of the elderly did suffer from diseases which were of minor importance to their daily living or were well regulated. These conditions were grouped into three categories according to the influence on balance and gait: no illness, illness which was not regarded to be balance related, and balance related illness. A balance related illness would include painful osteoarthritis in leg or lower back, foot deformities, dizziness, smaller sequelae after stroke, minor vascular disturbances in lower extremities, etc. Endocrine diseases, asthma, diabetes, well regulated hypertension and problems in the upper extremities were not regarded as balance related illnesses.

Self estimated health was scored on a 1–5 scale, with 1 being "very bad" and 5 being "very good". Balance confidence and fear of falling was scored using the Activity-specific Balance Confidence scale (ABC) [34]. The physical activity level of the participants was assessed by using the Physical Activity Scale for the Elderly (PASE) [35,36].

To record fall incidence, the subjects were given a fall diary which they were encouraged to keep. They were contacted and interviewed by phone after six and after twelve months. In this context a fall was defined as: "an event which results in a person coming to rest unintentionally on the ground or other lower level, not as a result of a major intrinsic event (such as stroke) or overwhelming hazard"[37]

### Test battery

Nine tests were selected for a test-battery to cover different aspects of physical performance related to fall risk. The tests ranged from specific tests of muscle strength to general tests on performance in combined tasks (table 3). In order to make the test-battery practical in a clinical setting, the following criteria were set: each test should be clinically applicable; total testing time should not exceed half an hour; conduction of the tests should not require a stationary setting. The selected tests had all been described and evaluated in scientific journals. In the following description the specific purpose and the test procedures are described for each of the nine individual tests.

**Table 3:** Test battery

	Test focus	Method	Form
1	Standing balance	"FICSIT-4 scale" + one leg eyes closed [24]	modified
2	Stepping ability	"Four Square Step Test" (FSST) [22]	original
3	General function	"Timed Up and Go" (TUG) [21,39]	original
4	Reaction time	Step reaction on visual cue [23]	modified
5	General leg strength	"Timed Stand Test" (TST) [40]	original
6	Dual task	Gait speed decrease in a "dual task" [42]	modified
7	Gait variability	Trunk acceleration autocorrelation [43]	modified
8	Gait cadence	Step cadence at gait speed 1.1 m/s [43]	modified
9	Vision	Visual acuity, contrast and field [47]	original

A listing of the nine tests selected for the test battery. The last column indicates whether the test was used in an original or a modified form. Detailed descriptions of the test procedures are provided in the method section.

#### 1. Standing balance

A test procedure was chosen which was used in the FICSIT-studies [24]. This procedure included the principles from the "Guralnik test", which is commonly used in the clinic [38]. This test addresses the participant's ability to adjust balance in response to the feedback from proprioceptors, vision and vestibular organs. Reducing the support area adds to the challenge of the test. The original procedure was expanded to avoid a ceiling effect by adding the task: "standing on one leg with eyes closed". The participant was asked to stand for 10 seconds with the feet in parallel, semi-tandem, and tandem position as well as to stand on one leg with eyes open and with eyes closed. Scores were given according to the ability to perform the tasks: Parallel refused ≈ 0.0; Parallel < 10 s ≈ 0.5; Semi-tandem < 10 s ≈ 1.5; Semi-tandem > 10 s and failed tandem ≈ 2.0; Tandem < 10 s ≈ 3.0; Tandem > 10 s, one leg < 10 s ≈ 4.0; One leg > 10 s ≈ 5.0; One leg eyes closed < 10 s ≈ 5.5; One leg eyes close > 10 s ≈ 6.0. The 0–6 score was converted into a 0–10 scale.

#### 2. Stepping ability

A test procedure called "Four Square Step Test" (FSST) was used for evaluating stepping ability [22]. During risky, balance challenging situations, the base of support must be altered by moving the feet to new positions. The ability to make these quick balance reactions by stepping forth, back and sideward is revealed by this test. Two sticks (height 2.5 cm and length 80 cm) were placed on the floor forming a cross. This cross indicated four squares (1, 2, 3, 4). The participants were asked to step as quickly as possible from one square to another in the order 1-2-3-4-3-2-1. They were asked to touch the ground with both feet in each square while facing in the same direction at all times. After a pre-trial, the faster of two trials was used for evaluation. A 0–30 s. score was used inversely for normalization.

#### 3. General physical function

"Timed Up and Go" test (TUG) is a widely used and a validated test for general physical performance in the elderly [21,39]. In this test the participant sat on a chair (height ≈ 46 cm.). A line was drawn on the floor three meters in front of the chair. The participants were asked to rise from chair, walk the three meters to cross the line, turn around, walk back, and sit down on the chair again. The time for this procedure was recorded by a stopwatch. The integrated factor of muscle strength and the ability to walk and turn around are evaluated by this test. A 0–20 s. score was used inversely for normalization.

#### 4. Reaction time

The step reaction time to a visual cue has been shown to be related to fall risk [23]. In a near-fall situation it is necessary to respond quickly to regain the balance and reaction time will give an insight of this ability. In our set-up, the participant was asked to stand in front of a wall at a distance of half a meter. A red and a green light were mounted on the wall at eye height and a red and a green footplate were placed 30 cm in front of the participant's feet 30 cm apart. The lights were alighted manually in a random order five times each, and the participant was asked to step onto the footplate of matching colour to the light as quickly as possible. The whole procedure was repeated with the foot plates placed at each side of the participant at a distance of 30 cm.

A step on the footplates triggered a pressure sensitive contact. This signal and the trigger time from the lights were recorded and the signal times were subtracted to find the reaction time. A mean reaction time from all trials was given. An inverse 0–2 s. score was used for normalization.

#### 5. General leg strength

Muscle strength is known to be related to falls risk [2]. A widely known clinical test for leg muscle strength called "Timed Stand Test" (TST) was used [40]. The time needed to rise from a chair ten times was recorded. The height of the chair was adjusted to the participant's leg length to maintain a knee angle at 90 degrees when sitting with the feet supported on the ground. The participant was instructed to rise and sit as fast as possible, and time taken for this was recorded using a stopwatch. A 0–60 s. score was used inversely for normalization.

#### 6. Dual task – gait automation

Walking should be an automated function which should not require much attention and it should be possible to perform a cognitive task while walking. However, it can be challenging to perform two tasks at the same time (dual task) if attention is needed in both tasks. Elderly fallers probably have a less automated gait and this explains why they seem to walk slower when performing a dual task [41]. To evaluate the dual task capacity of the participants a modified "Walking and Counting test" was used [42]. The participant was asked to walk a ten meter distance as quickly as possible. Then the same task was performed while now counting backwards in a 3-step sequence from 80. The walking time was recorded by a stopwatch, and the decrease in speed was given in percent. A 0–200 % score was used inversely for normalization.

#### 7. Gait variability

Walking is a challenging task, in which successful motor planning and fine tuned postural control are required to produce a smooth gait pattern. To reveal inadequacy in these matters, different gait measures can be used. During walking, the reaction forces from the floor are reflected in the trunk. An accelerometer placed at the lower back would move up and down, from side to side, and forward at alternating accelerations according to these forces. The recording of these alterations in acceleration offers a means of quantifying the gait. Measures on temporal stride-to-stride variability in the gait has proven to be predictors of fall risk [16]. By using accelerometry, even more information on the gait pattern is recorded, and a variability in the acceleration pattern between strides will be an indicator of the gait characteristics [43].

In this study the gait characteristics were measured by a tri-axial accelerometer placed at the participant's lower back at the L3 segment. Data from the accelerometer were stored in a portable data-logger carried behind the participant by the investigator. The participant was asked to walk a 14 meter distance on a flat floor. A trigger signal was manually activated when passing two markers on the floor. These markers were ten meters apart, and the participant would start and stop walking two meters before and after the respective markers. In this way a steady state gait for ten meters could be evaluated. The walking sequence was repeated six times at different speeds, – twice at individual preferred speed, twice at fast speed, and twice at slow speed. The raw data from the accelerometer were low-pass filtered at 50 Hz once in the forward and once in the reverse direction. The data were re-oriented to a vertical-horizontal plane for each gait speed as proposed by Moe-Nilssen [44]. Furthermore, an unbiased autocorrelation of the anterior-posterior accelerations was performed for each gait sequence which represented approximately eight strides [45]. The autocorrelation for a cyclic signal will produce peaks equivalent to the periodicity of the signal. The amplitude of the peak representing two phase shifts will relate to the variability between the strides. An autocorrelation coefficient of 1.0 would indicate that there is no variability between the gait strides at all, whereas a smaller coefficient would reflect a larger variability. The autocorrelation coefficients were averaged for the six different gait sequences. An autocorrelation score between 0.5 and 1.0 was used for normalization.

#### 8. Gait cadence

Gait speed has been seen as an indicator of fall risk [46]. Gait speed is a product of step length and cadence, and more detailed information might be gathered from recordings of the cadence. Step time was estimated from the interval between autocorrelation peaks given by the accelerometer measures, and this step time was inverted into a cadence given for each gait speed. As cadence increases with increasing gait speed the cadence was normalized to 1.1 m/s [45]. The cadence was furthermore normalized (to a body height of 1.65 m) by the square root of the height, as cadence is inversely proportional to the square root of body size [43]. A 1–3 steps/s. score was used inversely for normalization.

#### 9. Vision

Impaired vision is an important and independent risk factor for falls [18]. It is necessary to be able to see changes in the ground surface or obstructions in the walking path in order to plan and adjust the postural control in a feed-forward manner. Three tests were chosen to assess the vision as a feed-forward means for planning the gait: a. Visual acuity was assessed by using poster constructed for this purpose (Landolt "C" Translucent chart for 3-meter testing cat.no.2206, Precision Vision^®^, IL, USA). It was placed at a three meter distance in a light condition at approximately 400 lux. The participant was tested binocularly wearing normal glasses for walking. The test log-scores were converted into a rank scale: = 0.0 ≈ Normal vision (3 points); 0.1 – 0.4 ≈ Subnormal (2 points); 0.5 – 0.9 ≈ Weak sight (1 point); > 1.0 ≈ Very weak sight (0 points). b. A contrast sensitivity test was used to assess the participant's ability to detect contrasts (Pelli-Robson Contrast Chart 4 K, Clement Clark Int. Ltd., Essex, UK). The log contrast sensitivity scores were converted into a rank scale: ≥ 1.8 ≈ Normal (2 points); 1.36 – 1.8 ≈ Subnormal (1 point); ≤ 1.35 ≈ Weak (0 points). c. The visual field was tested using a confrontation test a.m. Donders [47]. The test was carried out for one eye at a time in the horizontal, the 135°, and the vertical plane. The performance was scored in ranks of 0 – 2 for each direction: > 60° ≈ Normal (2 points); 30 – 60° ≈ Reduced (1 point); < 30° ≈ Very reduced (0 points). A sum of these score ranged from 0 to 12, which again was ranked in three categories: 12 ≈ Normal (2 points); 7 – 11 ≈ Reduced (1 point); ≤ 6 ≈ Very reduced (0 points). Data from these three tests on vision were added and presented as a common 0–7 score, which was normalized into a 0–10 scale.

### Data Analysis

Signal processing of the accelerometer signals and the trigger signals on reaction time was performed in MatLab (ver. 6.1, MathWorks Inc.). Data organization was done in Excel (2002, Microsoft Corp.) and the statistics were conducted in SPSS (ver. 12.0, SPSS Inc.). Power calculation was done in an online calculation from the Department of Statistics, UCLA [48].

To compare group characteristics and test scores in the fallers and non-fallers group, Student's t-tests (for nominal data) and Mann-Whitney U tests (for ordinal data) were used. Binary and backward stepwise logistic regression was used to evaluate the predictive capability of the test battery and the contribution to the prediction of individual tests. To be able to evaluate a common score of the test battery score in relation to fall risk the original scores from the individual tests were converted into 0–10 scales. A conversion was chosen for each test which allowed for the minimum and maximum scores. In order to let higher values present better performance, some test scores had to be reversed. The normalized test scores were averaged into a common test battery score. The predictive rates of the test battery in relation to the variable "faller" and "non-faller" was evaluated at a selected optimal cut-off value (so called crude discrimination rates).

The one-sided power of the study was estimated in relation to a relevant mean difference in test battery score of 0.5 equal to a 5% difference. Furthermore the least critical difference was estimated for a power of 80%.

Chi-square test and one-way ANOVA with post-hoc tests were used when the data was evaluated according to balance-related illness.

## Competing interests

The author(s) declare that they have no competing interests.

## Authors' contributions

All authors participated in the design of the study. TS was involved in the overall organisation and coordination. OS supervised and contributed in clinical aspects of the study. HCH was involved in recruitment of participants and supervised the contacts. MV supervised the technical methodology and was involved in the data analysis and drafting of the manuscript. UL designed the study, carried out the testing and data analysis and was main author of the manuscript.
